# Fiberglass and Other Flame-Resistant Fibers in Mattress Covers

**DOI:** 10.3390/ijerph19031695

**Published:** 2022-02-01

**Authors:** Jeff Wagner, Jefferson Fowles, Tracy Barreau

**Affiliations:** 1 Environmental Health Laboratory Branch, California Department of Public Health, 850 Marina Bay Parkway, Richmond, CA 94804, USA; 2 Environmental Health Investigations Branch, California Department of Public Health, 850 Marina Bay Parkway, Richmond, CA 94804, USA; jeff.fowles@cdph.ca.gov (J.F.); tracy.barreau@cdph.ca.gov (T.B.)

**Keywords:** flame retardants, fiberglass, mattresses, exposure assessment

## Abstract

Public complaints have raised concerns that some mattresses in the current marketplace may be potential sources of airborne fiberglass. Although mattress foam is often marketed as chemical-free, their cover compositions are not as well understood by the general public. To fill these basic information gaps, the covers of four newly purchased mattresses were sampled and analyzed using polarized light microscopy, SEM-EDS, and FTIR microspectroscopy. Two of the mattress covers contained over 50% fiberglass in their inner sock layers. Up to 1% of the fiberglass had migrated to adjacent fabric layers, representing a potential risk of consumer exposure if the zipper on the outer cover is opened. The observed fiberglass fragments had calculated aerodynamic diameters ranging between 30 and 50 µm, suggesting they are potentially inhalable into the nose, mouth, and throat, but are likely too large to penetrate deeper into the lungs. No fiberglass was observed on the brand new mattresses’ outer surfaces. Synthetic fibers also present in the sock layers were consistent with flame resistant modacrylic containing vinyl chloride and antimony. The use of fiberglass and other chemicals in mattress covers poses a potential health risk if these materials are not adequately contained. The apparent non-inclusion of mattress covers in chemical-free certifications suggests that further improvements are needed in mattress labeling and education of consumers.

## 1. Introduction

Fiberglass, sometimes also referred to as man-made vitreous fibers (MMVF), is a known respiratory, skin and eye irritant and an asthmagen [1]. In occupational settings, fiberglass exposure has been found to correlate with recurrent chest infections and pulmonary fibrosis [2,3]. Fiberglass fibers can vary in diameter, length, and chemical composition, but are predominantly amorphous (non-crystalline) mixtures of oxides of silicon, calcium, and other metals. Common commercial uses for fiberglass include insulation and fire protection [4].

Prompted by historical concerns that chronic inhalation of these fibers could pose lung cancer risks, the International Agency for Research on Cancer (IARC) reviewed the literature on at least two occasions, and concluded in 2001 that the evidence for carcinogenicity from fiberglass exposure met the criteria for Group 3 (unclassifiable as to human carcinogenicity) [4]. This conclusion was supported by a subsequent review of the occupational epidemiology literature, including case–control, cohort, and meta-analyses, in 2011 [5]. The carcinogenicity of a given fiberglass fiber depends on both its inhalability (aerodynamic size) and its biopersistence (durability and clearance rate from the lung) [6].

Several anecdotal reports and numerous public complaints have raised concerns that some mattresses in the current marketplace may be potential sources of airborne fiberglass exposures. Children and infants represent a special potential risk group, both due to their increased susceptibility and the possibility that children may play or jump on beds. In one case investigated by CDPH in 2021, a 6-year old child was found to have persistent skin and respiratory irritation linked to the suspected leakage of fiberglass from a mattress purchased in 2018. The resulting clean-up of the home included disposal of the mattress, carpet, and clothing items from which the fiberglass fibers could not be removed by cleaning. A search of the Consumer Product Safety Commission website found 128 complaints from 1 January to 31 December 2020 linked to fiberglass exposure in several different brands of mattresses, and news media have reported on a growing number of relevant health complaints [7,8].

The inclusion of flame retardants and flame-resistant fibers in mattresses is primarily driven by California and US flammability regulations, despite the fact that their use in products poses a potential health risk [9]. Changes in California furniture flammability testing rules have shifted emphasis from the foam to their covers [10], so the prevalence of these additives in current mattress covers is an important unknown. “Certi-PUR-US” is an industry-based certification program that designates foam products to be free of heavy metals, PBDEs, TDCPP or TCEP (“Tris”) flame retardants, as well as numerous flame-retardant additives [11]. Consumers are likely to believe CertiPUR-US-certified mattresses have undergone rigorous testing and are free of hazardous substances. However, the certification and testing do not appear to include the mattress covers.

The aim of this project was to fill basic information gaps about the physical/chemical nature of fibers in new mattress covers by analyzing them with microscopy and microspectroscopy. We purchased four new mattresses including one child mattress, and sampled and analyzed their covers using polarized light microscopy (PLM), scanning electron microscopy with X-ray energy dispersive spectroscopy (SEM/EDS), and Fourier transform infrared (FTIR) microspectroscopy. We report on the basic structure of these covers, the size and chemical characteristics of the detected fiberglass materials, and the additional flame-resistant fibers observed in the analyses.

## 2. Materials and Methods

The mattresses were purchased and delivered from common online sales outlets in April and May of 2021. The four purchased mattresses were a Sealy Essentials Joyfulness 8.5” twin (Referred to hereafter as mattress FG-1), a Modway Aveline 6” twin (FG-2), a Zinus Green Tea 6” memory foam twin (FG-3), and a Graco crib & toddler deluxe (FG-4). The polyurethane foam in all of these products was advertised as being certified “Certi-PUR-US” (see Supplementary Materials Table S1).

The constituent components of the four mattress covers were each subsampled by cutting through each observed layer with a clean, stainless-steel scalpel, then collecting the pieces into small, labeled, zipper-lock bags. Each piece was subsampled through its entire layer depth, with square cross-sectional areas of approximately 1 cm^2^. No foam was tested in this work.

Sample preparation and analysis was conducted using an in-house standard operating procedure for analyzing the fibrous content of bulk materials, based on EPA 600/R-93/116 [12]. A combination of subsample PLM and whole-sample stereozoom analyses was used to determine component fractions via calibrated visual estimation and species-specific optical properties, supplemented with SEM-EDS and ash analyses for confirmation. The reporting limit for this procedure is 1%, and species detected at less than this concentration have been reported as trace (<1%). Although this SOP is oriented towards determining asbestos content, it also includes specific procedures for identifying fiberglass and synthetic fibers. CDPH is accredited in the use of this SOP, participates in quarterly proficiency testing, and maintains a library of over 300 consumer and building material reference samples.

All subsamples were initially inspected and documented with a Leica S8APO reflected-light stereozoom microscope and DFC420 digital camera (Leica, Wetzlar, Germany) at 6.3–80× magnification. This work focused on the non-foam cover components as the most likely sources of fibers, but the foam components were archived for potential future analyses.

PLM was conducted using a Nikon E-600POL and DS-Ri1 digital camera with DS-Ri1-U2 controller (Nikon Instruments, Melville, NY, USA) at magnifications of 40×, 100×, 200×, and 400×. Slide mounts were prepared with 1.680 refractive index oil (Cargille Laboratories, Cedar Grove, NJ, USA) which was certified annually. All images in this report were acquired using crossed polarizers and a full-wave (red one) compensator. Under these imaging conditions, fiberglass can be positively identified by an absence of interference colors and total extinction (indicating an isotropic index of refraction and amorphous material), in conjunction with an elongated aspect, blunt fiber end terminations, and a uniform cross-section [13]. Fiberglass often possesses a straight, rod-like morphology, but some varieties of silicaceous fibers (more often called mineral or rock wool) also exhibit curved fibers. Imaging was also conducted using plane polarized light, crossed polarizers with no compensator, and dispersion staining conditions, respectively, to check for other components.

When no fiberglass was detected in any samples for a given mattress cover, all constituent samples were ashed in a muffle furnace at 485 °C for 3 h. This procedure removes any polymeric, combustible, and lower-boiling point components, leaving only non-volatile remnants such as fiberglass, other minerals, and ash.

A fiberglass standard (NIST Standard Reference Material SRM 1866a, Gaithersburg, USA) was analyzed as a reference. This SRM contains a range of glass fiber types and thicknesses ranging from 1–10 µm.

A blank sample containing non-fiberglass synthetic fibers was prepared and analyzed along with these samples to check for any fiberglass contamination in the laboratory. Analysis of this blank yielded no fiberglass fibers. This blank also contained crystalline silica (quartz) particles and was used as a reference for the presence of any quartz in the samples.

Supplemental SEM/EDS was conducted using an FEI XL30 Environmental SEM with Noran System 7 EDS (Thermo Fisher Scientific, Madison, WI, USA) and SEMView 8000 electronics (SEMTech Solutions, North Billerica, MA, USA). Bulk samples were prepared on standard aluminum SEM stubs with double-sided, carbon adhesive tabs and were carbon-coated to minimize charging. Limited “tape lift” samples were also collected from the outer covers using one SEM stub/tab per mattress. Samples were imaged at 20 kV and 20–2500× using a tungsten source and an atomic number-sensitive back-scattered electron (BSE) detector for rapid screening differentiation of non-carbonaceous materials.

Limited synthetic fiber and flame retardant identification was performed using a Nicolet iN-10MX FTIR Microscope with Omnic Picta software and a cooled MCT-A detector (Thermo Fisher Scientific, Madison, USA). Spectra were acquired from fiber strands with 4 cm^−1^ spectral resolution from 700–4000 cm^−1^ in transmission mode mounted on a standard 3-hole slide. Identifications of component mixtures were assigned manually with the aid of quantitative FTIR spectral searches and in-house and commercial libraries of over 100,000 spectra. The estimated limit of detection for flame retardants and other minor constituents of polymer materials is in the order of 1% [14].

## 3. Results

### 3.1. Main Components of Mattress Covers

**Figure 1:**
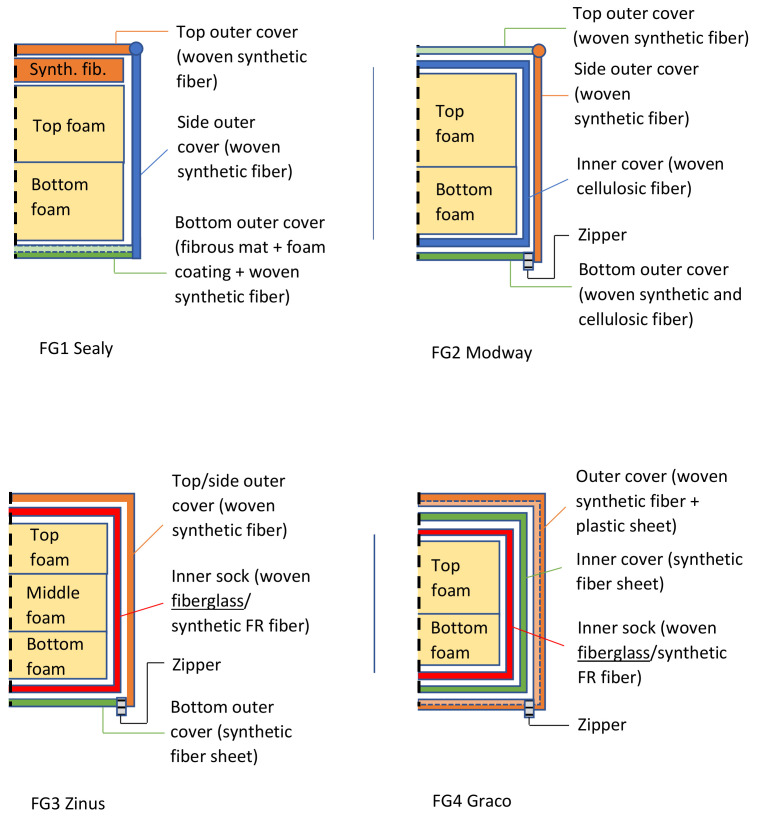
Main components of each tested mattress and observed compositions.

**Figure 2:**
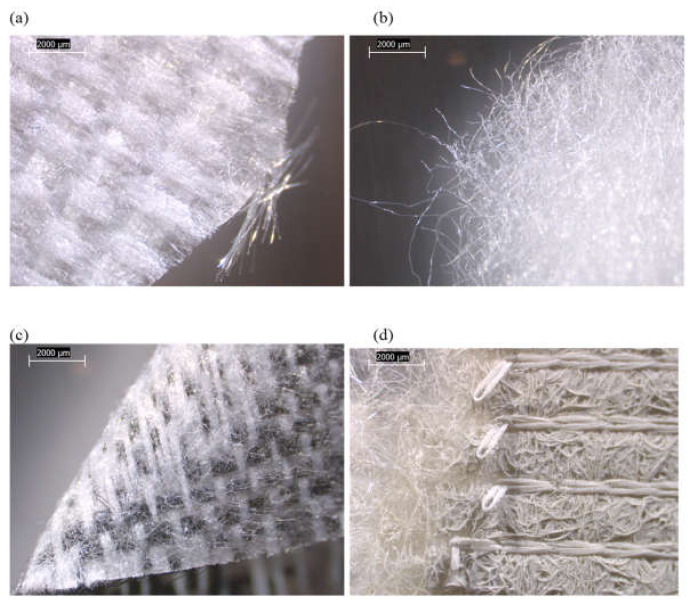
Low-power reflected-light stereozoom microscope images of FG-1 mattress subsamples acquired at 10×. (**a**) Synthetic, woven outer cover material from top side of mattress. (**b**) Synthetic, fibrous mat beneath outer cover material from top side of mattress. (**c**) Synthetic, woven outer cover material from side of mattress. (**d**) Outer cover material from bottom of mattress with fibrous mat, foam coating, and woven fibers.

**Figure 3:**
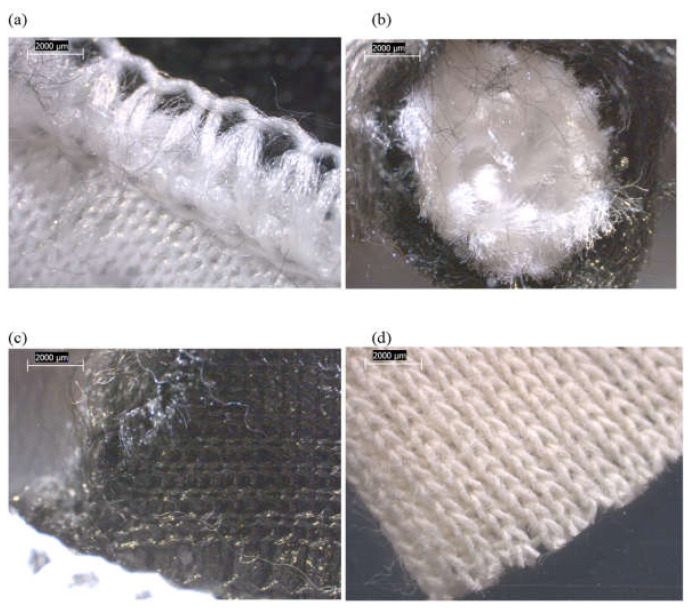
Low-power reflected-light stereozoom microscope images of FG-2 mattress subsamples acquired at 10×. (**a**–**c**) Three sections of the synthetic, woven outer cover materials, including fiber-filled bead (**b**). (**d**) Inner cover material with natural, woven fibers.

**Figure 4:**
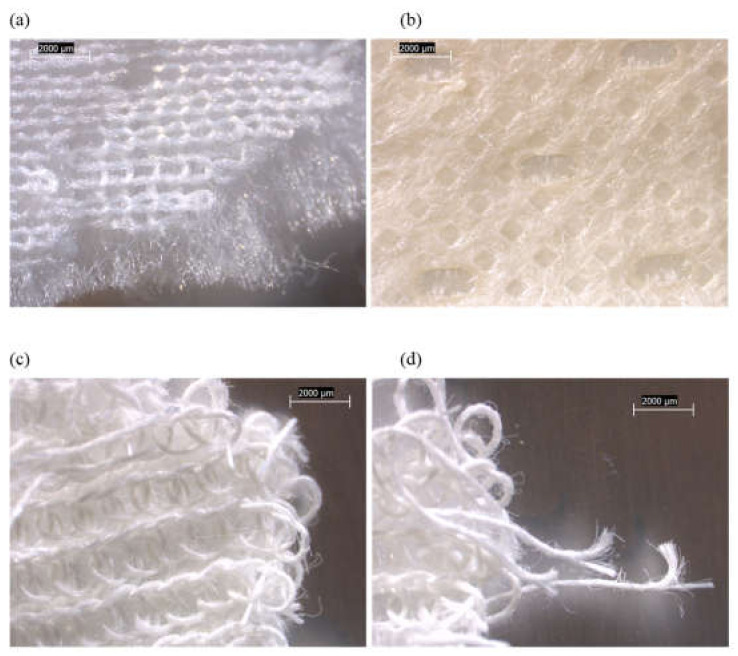
Low-power reflected-light stereozoom microscope images of FG-3 mattress subsamples acquired at 10×. (**a**) White, synthetic, woven outer cover material from top side of mattress. (**b**) Beige outer cover material from bottom side of mattress consisting of synthetic fibers in polymeric binder. (**c**) Inner sock material, showing woven threads, each uniformly wrapped in white synthetic fibers. (**d**) Same as (**c**), but inner fiberglass core revealed inside each thread.

**Figure 5:**
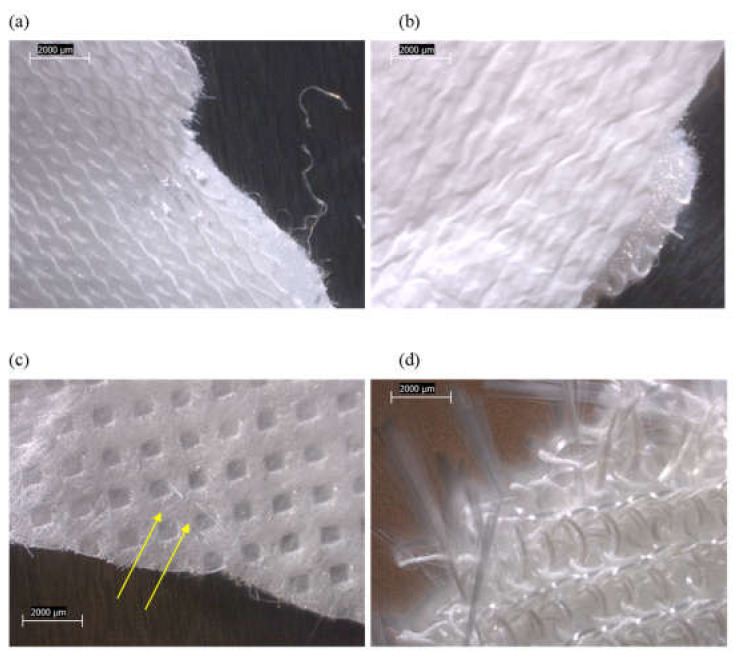
Low-power reflected-light stereozoom microscope images of FG-4 mattress subsamples acquired at 10×. (**a**) White, synthetic, woven outer surface of outer cover material. (**b**) White, synthetic inner plastic sheet surface of outer cover material. (**c**) Inner cover material consisting of synthetic fibers in polymeric binder, and short straight fiberglass fibers sticking into surface (indicated by arrows). (**d**) Inner sock material, showing woven threads consisting alternately of white synthetic fibers and shiny/clear fiberglass bundles.

The main components of each tested mattress cover and their observed compositions are summarized in Figure 1 and Table S2. Three of the four mattresses (FG-2, -3, and -4) possessed zippers on their outer covers. The various mattress cover components of FG-1 and FG-2 were mixtures of synthetic fibers, mostly in the form of woven materials (Figure 2 and Figure 3). The mattress FG-1 additionally contained fibrous mat layers beneath the top cover and inside the bottom cover, as well as a synthetic coating (Figure 2). FG-2 also contained cellulosic (natural) fibers. The outer cover of FG-3 on the back side of the mattress (Figure 4b) and the inner cover of FG-4 (Figure 5c) were both pre-formed sheets composed of organic binder and synthetic fibers, and the outer cover of FG-4 had a solid plastic sheet bonded to its inner surface (Figure 5b).

**Figure 6:**
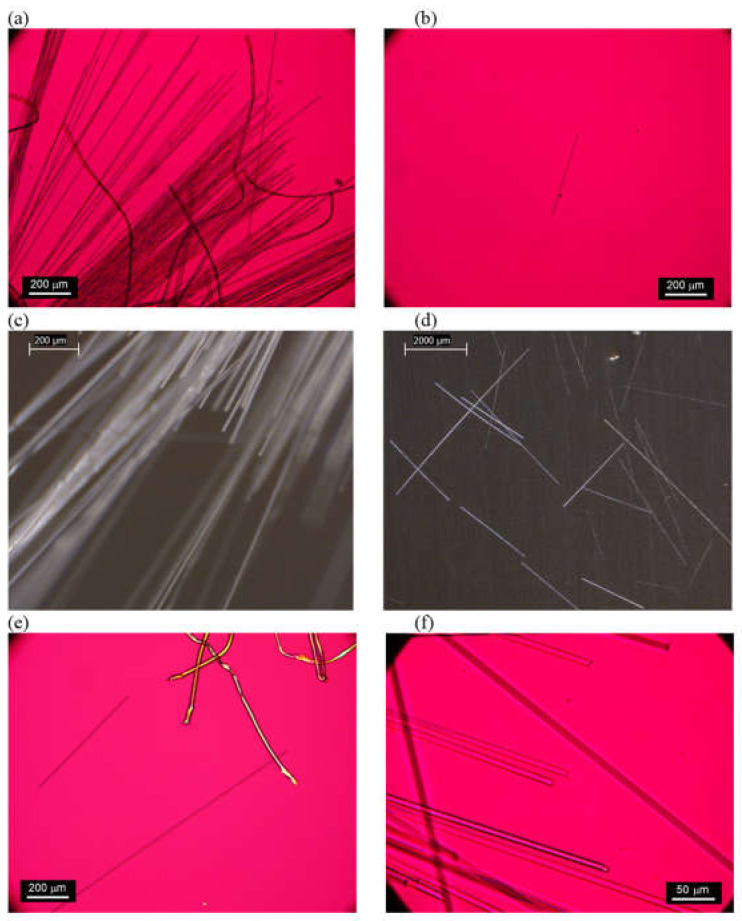
(**a**) Fiberglass (straight) in FG-3 inner sock material with synthetic fibers (wavy) (100× PLM). (**b**) Rare fiberglass fragment in FG-3 bottom, outer cover material (100× PLM). (**c**) Fiberglass in FG-4 inner sock (80× stereozoom). (**d**) Fiberglass shed onto foil surface after FG-4 inspection (10× stereozoom). (**e**) Fiberglass in FG-4 inner cover material with synthetic fibers (100× PLM). (**f**) Higher magnification of fiberglass morphology from FG-4 inner sock (400× PLM).

In addition to these polymeric cover materials, FG-3 and FG-4 both possessed inner “sock” layers that were accessible beneath a zipper in their outer covers. The sock compositions of both were dominated by straight bundles of fiberglass (Figure 4 and Figure 5 and Figure S1). PLM confirmed no fiberglass content in FG-1 or FG-2, but the positive detection of fiberglass for FG-3 and FG-4 (Figure 6 and Figures S2–S6). The fiberglass bundles in FG-3 and FG-4 were intermixed to different degrees with synthetic (polymeric) fibers.

Other than polymeric binders, the non-fibrous components of the tested materials were minimal, on the order of 1%. No crystalline silica (quartz) particles were observed in these samples by PLM.

### 3.2. Fiberglass Characteristics

In sample FG-3, each fiberglass bundle was completely encased in a synthetic fiber sheath layer such that no fiberglass was visible except at the cut ends, and the cloth maintained much of its structural integrity after being cut. This sock material was approximately 50% fiberglass by mass. In FG-4, however, bare fiberglass bundles were interwoven separately with less numerous woven synthetic fiber strands. This sock material was approximately 75% fiberglass by mass and was observed to visibly degrade and shed fiberglass pieces, especially where it was cut. Although cutting does not represent normal bed usage, the FG-4 sock’s degradation (Figure 6d) suggests a relatively lower structural integrity compared to sample FG-3.

The 400× PLM imaging revealed the fiberglass fibers in FG-3 and FG-4 to be fairly uniform, straight, and 5–10 µm in thickness (Figure 6f and Figure S6), consistent with previous surveys of fiberglass in textiles [1] and the thickest fibers in the NIST SRM mixture. The longest intact fiberglass bundles were very long, comparable to the sizes of subsamples removed for analysis (3–5 cm), and may have been even longer in the original material. However, the numerous fiberglass fragments shed from FG-4 during inspection were much shorter, many approximately 2 mm long. The shortest fiberglass fragments observed at 400x PLM were approximately 50 µm long.

**Figure 7:**
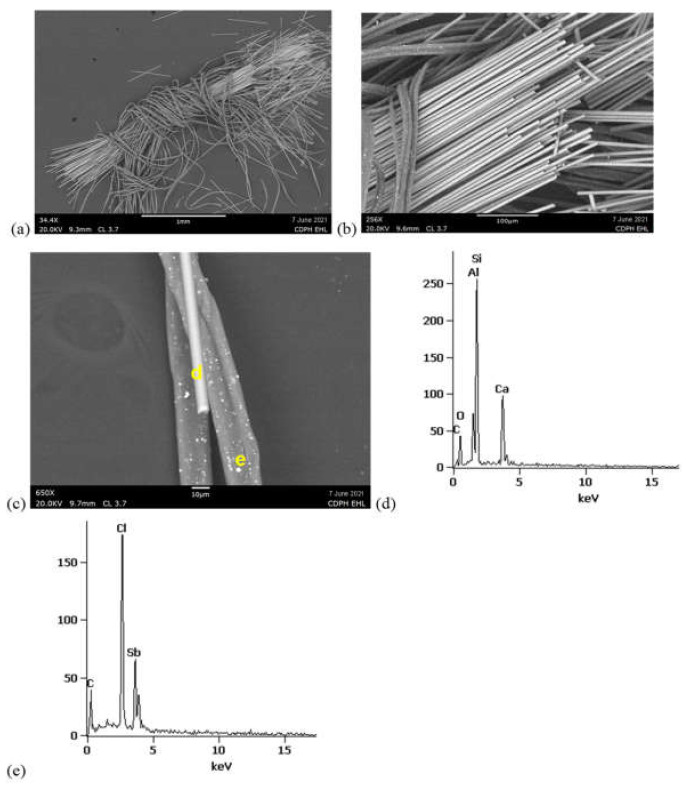
SEM-EDS from FG-3 inner sock. (**a**, **b**) Cut bundle of fiberglass wrapped in synthetic fibers. (**c**) One fiberglass fiber and two synthetic fibers with characteristic bright regions. (**d**) EDS acquired from fiberglass region marked in (**c**) showing Si, Al, and Ca. (**e**) EDS from bright spot in synthetic fiber marked in (**c**) exhibiting Cl and Sb.

**Figure 8:**
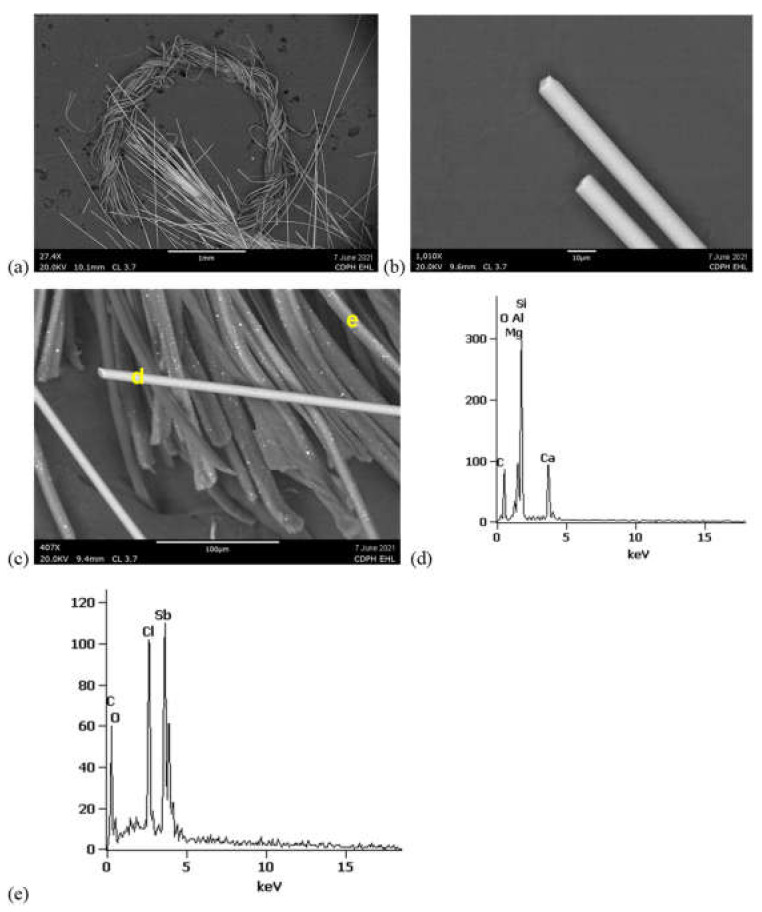
SEM-EDS from FG-4 inner sock. (**a**) Cut bundle of fiberglass and separate bundle of synthetic fibers. (**b**) Single fiberglass fibers with blunt ends and 8 um thickness. (**c**) Fiberglass fibers and synthetic fibers. (**d**) EDS acquired from fiberglass region marked in (**c**) showing Si, Al, and Ca. (**e**) EDS from bright spot in synthetic fiber marked in (**c**) exhibiting Cl and Sb.

SEM/EDS analyses of the inner sock layers from samples FG-3 and FG-4 confirmed the same fiberglass morphology as was observed with PLM and 5–10 um-thick fibers (Figure 7 and Figure 8). The fiberglass in both samples exhibited the typical mineral fiber composition of primarily silicon (Si), aluminum (Al), and calcium (Ca).

Analyses of the cloth layers adjacent to the fiberglass layers in samples FG-3 and FG-4 revealed instances of loose fiberglass adhered to them, likely due to migration from the inner socks. For FG-3, only a trace amount of fiberglass (5 fibers) was found adhered to the bottom outer cover (Figure 6b), and no fiberglass was observed in the top cover. Adhered fiberglass fragments were much more commonly found adhering to the inner cover of FG-4 (Figure 5c and Figure 6e), representing approximately 1% of the material by mass.

No fiberglass was observed adhered to the outer cover of FG-4, however. It is possible that the migration of fiberglass from the inner cover was prevented to some extent by the solid polymer sheet sublayer of the outer cover.

### 3.3. Other Flame Resistant Fibers

The ashing of samples from FG-1 and FG-2 resulted in near-total volatilization of most components at 485 °C, confirming they did not possess major fiberglass content like FG-3 and FG-4. Some non-flammable fibers remained in FG-1, however. The fibrous mat material from the top and bottom of mattress FG-1 survived 485 °C, with little change in appearance (Figure S2c,f), and the fiber coating in the bottom outer cover also remained (Figure S2f). Only charred, shriveled fiber remnants from the inner cover survived the ashing of FG-2 (Figure S3c,d). The limited FTIR of fibers from FG-1 variously matched polyester, polypropylene, and viscose (Rayon), respectively (Figure S7). Additional small peaks matching antimony trioxide and silicic acid were inconclusive, but may help explain these fibers’ persistence at high temperatures.

**Figure 9:**
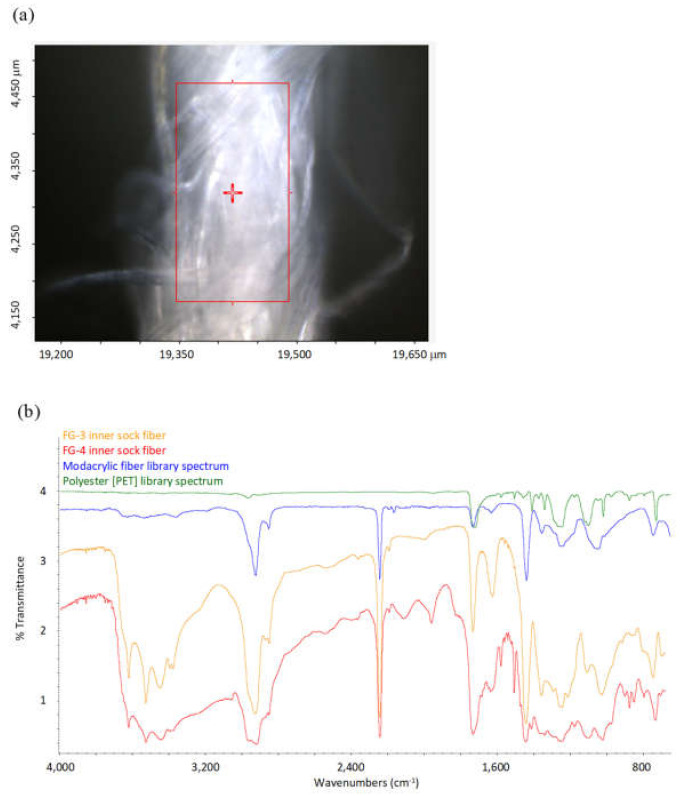
(**a**) FTIR microscope image of analyzed area of 200 um thickness, synthetic fiber bundle from inner sock material in FG-4. (**b**) FTIR microspectroscopy results (red spectrum) from (**a**). Also shown is a spectrum from the inner sock of FG-3 (orange). Both are good matches with the library spectra for modacrylic fibers containing vinyl chloride and antimony trioxide additives (blue). In addition, the synthetic fibers from FG-3 contain minor peaks matching PET (green).

SEM/EDS BSE of synthetic fibers in both FG-3 and FG-4 revealed them to be carbonaceous, with sub-micron, brighter inclusions characteristic of manufactured polymers with inorganic additives (Figure 7 and Figure 8). EDS acquired from these bright regions revealed enriched chlorine (Cl) and antimony (Sb), consistent with a flame-retardant product with metal synergists [15,16,17]. FTIR analyses of these synthetic fibers from the inner sock layers of FG-3 and FG-4 matched library spectra for modacrylics with vinyl chloride and antimony trioxide flame-retardant additives (Figure 9). Although these FTIR peaks were also small, the detection of Cl and Sb with SEM-EDS confirms the presence of flame retardants in the FG-3 and FG-4 fibers. Additional peaks in the FTIR spectra from FG-3 suggest they were blended with PET (polyester terephthalate) fibers. In general, most of the detected polymer fiber types in the covers of FG-1, FG-3, and FG-4 were listed on their tags, but with no flame-retardant specification (Table S1).

## 4. Discussion

Our investigation confirmed the presence of fiberglass in two of the four tested mattress covers. The fiberglass was evident in layers that were accessible beneath outer zipper layers (Figure 1), presenting a relatively easy route of exposure. The presence of fiberglass was not disclosed on the labels of the FG-4 mattress, leaving consumers unaware of its presence. Fiberglass was disclosed on the label of FG-3, though not in the most prominent flammability section (“law label”), which is required to specify filling materials only [10].

At minimum, any exposure to fiberglass represents a potential dermal irritation, as well as an eye irritation hazard [4,5]. However, the potential of fiberglass to additionally represent an inhalation hazard depends on whether the material is aerosolizable and the range of potential fiber widths and lengths [4]. As described in the Results, both FG-3 and FG-4 contained very long fiberglass fibers, but these were observed to fragment into smaller pieces when cut or disturbed, particularly in the case of FG-4.

The effective aerodynamic size, d_a_, for fibers in an oriented fluid flow can be calculated using an assumed density of ρ_f_ = 2.5 g/cm^3^ [18], and the following expression [19,20]:

d_a_ = W × (9/4 × ρ_f_/ρ_0_ × [ln(2L/W) − 0.807])^0.5^ (1)

where W = fiber diameter, ρ_0_ = unit density, and L = fiber length. For the smallest observed fiberglass fragments (50 µm long and 5 µm wide), d_a_ = 30 µm. For the commonly observed, longer fiberglass fragments (2 mm long and 10 µm wide), d_a_ = 50 µm.

Standard inhalability curves predict substantial head airway (nose, mouth, pharynx, and larynx) deposition for particles with approximately da = 10–100 um, while standard thoracic and respirable curves predict enhanced tracheobronchial and alveolar lung deposition for d_a_ < 10 µm and 2.5 µm, respectively [21]. These curves suggest that the observed fiberglass fragments from these covers are potentially inhalable into head airways, but are likely too large to penetrate deeper into the chest or lungs. As such, they are potentially a nasal and throat irritant and asthma trigger, but unlikely to be a lung hazard. Note that exposure to extreme environments, solvents, or mechanical damage during manufacture or product aging may alter fiberglass friability, or further reduce fragment sizes from those observed here.

FTIR micro-spectroscopy determined that the synthetic fibers in the fiberglass socks were additional sources of flame-resistant fibers (e.g., modacrylics), and SEM/EDS confirmed they also contained halogenated compounds and metal synergists (antimony trioxide). It is not yet known to what degree these flame-retardant additives may represent additional risks thorough dermal, oral, or inhalation exposure routes [17,22]. However, many of these compounds are prohibited under the CertiPUR-US certification of the foam, which may lead consumers to believe they are not present in the mattress.

Similarly, flammability tags attached to the mattress covers containing fiberglass and antimony-containing fibers (FG-3 and FG-4) contained statements such as “100% polyurethane foam pad”, suggesting they were referring to the foam cores only. California law currently requires the labeling of flame retardants in specific product types if they represent >0.1% of the product mass [17], but exempts some mattress types and their non-foam components [10,22]; flame retardants in non-foam mattress components must be labeled if they are for toddlers or infants [23]. Accordingly, the only mattress in this study that possessed such a label (FG-4) was a crib mattress, but it listed “no added flame retardant chemicals”, despite the presence of fiberglass and modacrylic fibers in the cover. In this regard, it is unclear whether fiberglass is considered a flame-retardant chemical using current guidelines [24,25]. In general, the possibility that none of these mattress cover contents are included in current chemical-free certifications suggests that improvements are still needed in mattress certifications, labeling, and the education of consumers.

This work was limited to four mattress covers obtained from the current marketplace. Future analyses of other mattress brands and models would be useful to determine whether these findings apply to mattress covers in general. The observations of inner sock friability during the laboratory analyses are suggestive of differential fiberglass exposures between brands and sock designs. More systematic experiments of fiberglass shedding under realistic environments and mechanical stresses are needed, and could be combined with standard methods to measure fiberglass fragments in air [26], dust [16,27], or eye or nose mucous samples [28,29]. This work focused on the presence of fiberglass and other flame-resistant fibers in mattress covers, but future analyses of the associated foam materials would provide a useful complement to this information. The extent to which our results raise a generalizable issue related to fiberglass exposure from other consumer products that meet flammability standards is unknown, but worthy of investigation.

## 5. Conclusions

We determined the basic structures and compositions of select, new mattress covers using various microspectroscopy analyses. Fiberglass was observed in two of the four covers, including potentially inhalable fiberglass fragments that pose a health risk if the covers are opened by consumers. Further, undisclosed chemical additives were observed, including modacrylics containing antimony and vinyl chloride, and these could present additional exposure and health risks. Although flame-resistant fibers are used in mattress covers to pass flammability regulations, their compositions are uncertain on labels that may describe the foam contents only. The apparent omission of mattress covers from the criteria for chemical-free certifications of mattresses suggests that improvements are needed in mattress labeling and the education of consumers.

## Data Availability

All data used to form conclusions are contained within the paper and Supplementary Materials.

## Supplementary Materials

The following supporting information can be downloaded at: https://www.mdpi.com/article/10.3390/ijerph19031695/s1, Table S1: Mattress labels, certifications, and cover fibers identified; Table S2: Summary of compositions determined from mattress cover analyses; Figure S1: Additional 10–80× images of FG-4 inner sock; Figure S2: PLM images of FG-1.; Figure S3: PLM images of FG-2; Figure S4: PLM images of FG-3; Figure S5: PLM images of FG-4; Figure S6: 400× PLM images of fiberglass morphology; Figure S7: FTIR from three FG-1 fibers.
