# Supplementary material for: Fiberglass and Other Flame-Resistant Fibers in Mattress Covers
Source: Int J Environ Res Public Health. 2022 Feb 1;19(3):1695. doi: 10.3390/ijerph19031695 (PMC8835476; doi:10.3390/ijerph19031695)
Supplement: Supplementary file 1 [file ijerph-19-01695-s001.zip › ijerph-1543338-supplementary.pdf]

Supplemental Materials

Fiberglass and other Flame-resistant Fibers in Mattress Covers

Jeff Wagner, Jefferson Fowles, and Tracy Barreau

(a) (b)

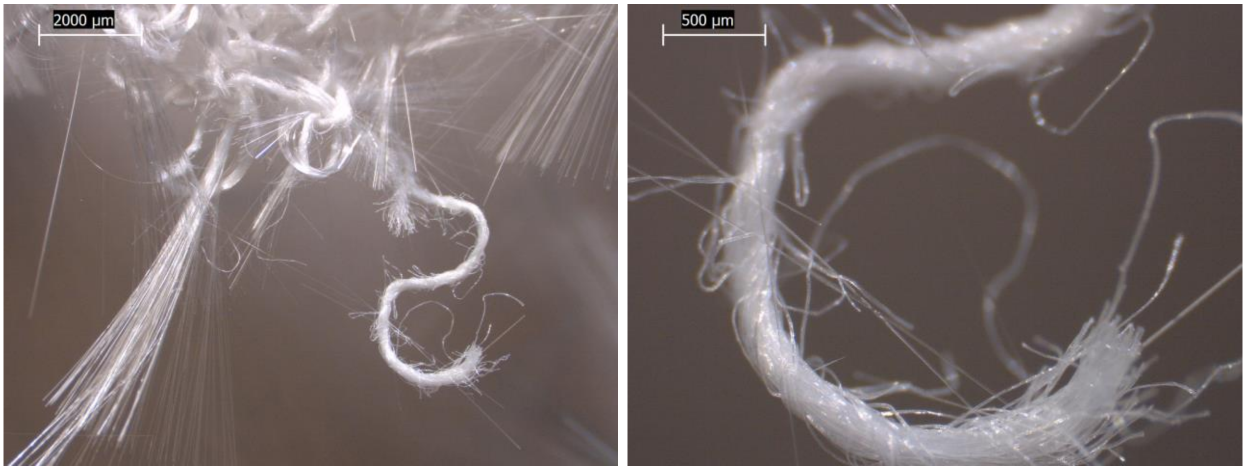

Figure S1. Additional 10x-80x images of FG-4 inner sock using low-power reflected light stereozoom microscope. a) straight fiberglass bundles and wavy synthetic fiber threads. b) higher magnification image of fibers in (a).

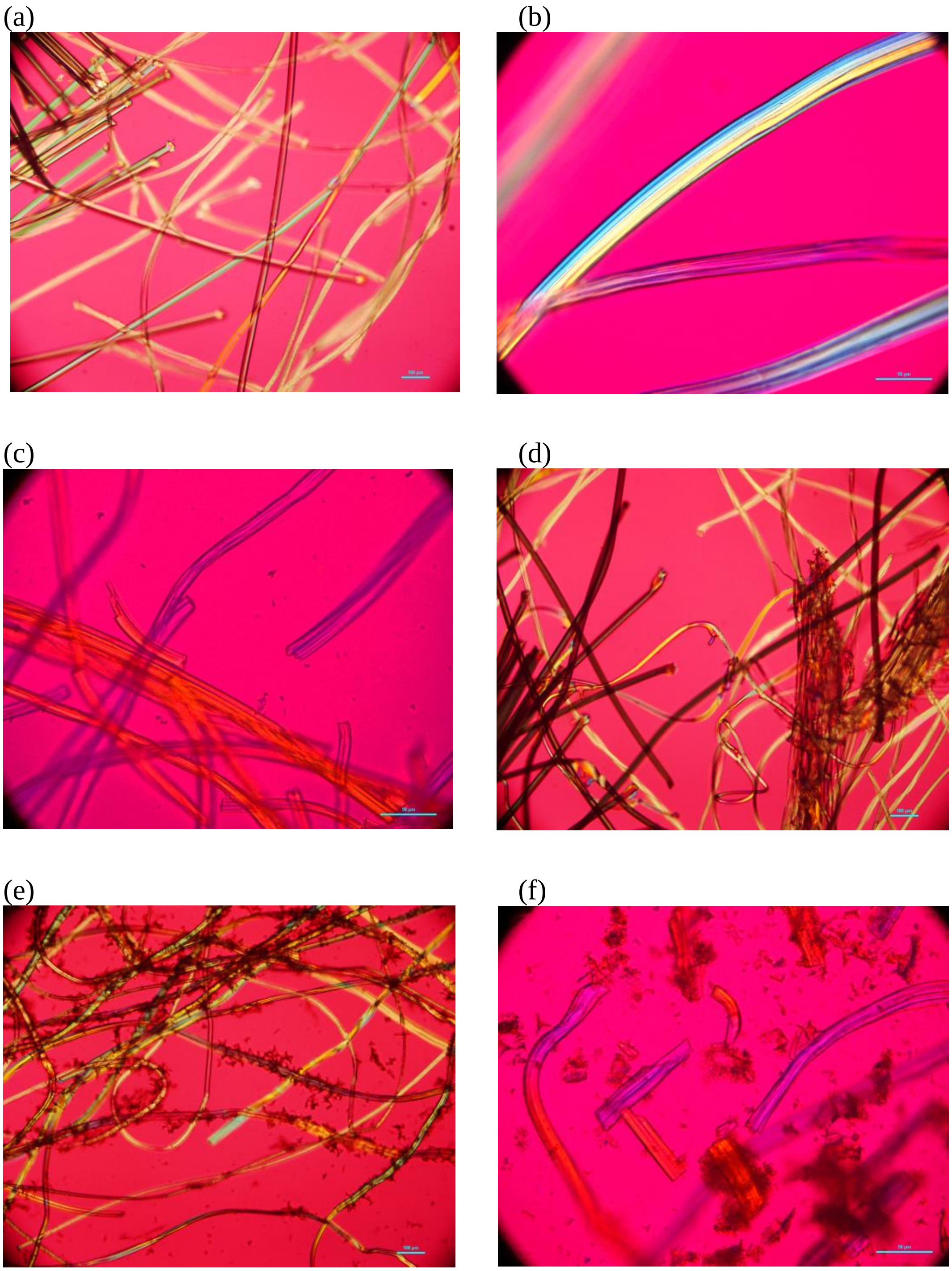

Figure S2. PLM images of FG-1. a) outer cover from top side of mattress showing mixture of birefringent synthetic fibers (100x). b) fibrous mat below top cover (400x). c) same sample as (b) after ashing at 485 C (400x). d) outer cover from mattress side showing mixture of birefringent synthetic fibers (100x). e) outer cover from mattress bottom showing mixture of birefringent synthetic fibers and coating (100x). f) same sample as (e) after ashing at 485 C (400x). The scale bars are 100 μm long (100x) and 50 μm long (400x).

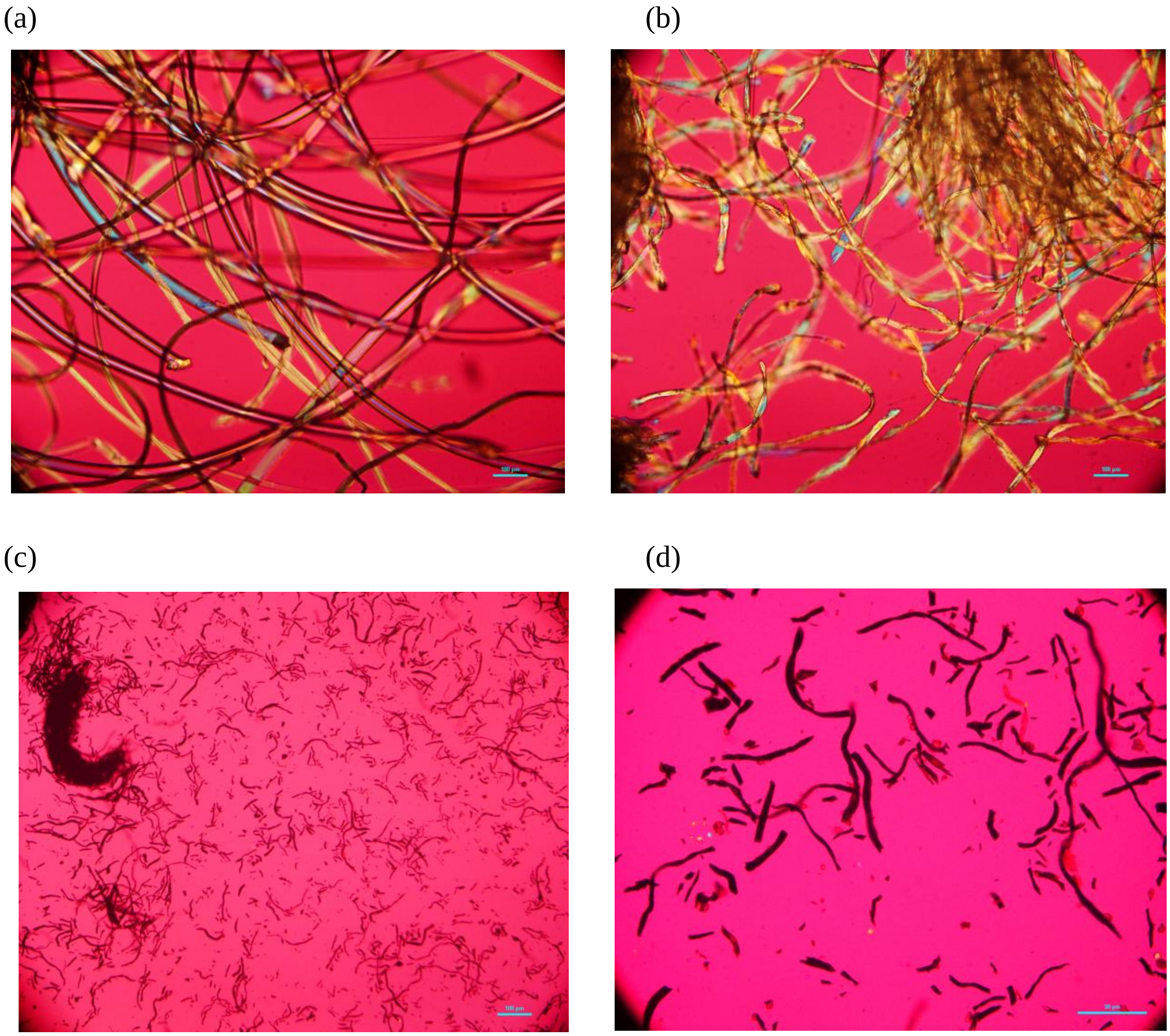

Figure S3. PLM images of FG-2. a) outer top cover material showing mixture of birefringent synthetic fibers (100x). b) inner cover material showing mixture of birefringent cellulosic fibers (100x). c) same sample as (b) after ashing at 485 C, showing charred fiber remnants (100x). d) same as (c) at 400x. The scale bars are 100 μm long (100x) and 50 μm long (400x).

(a) (b)

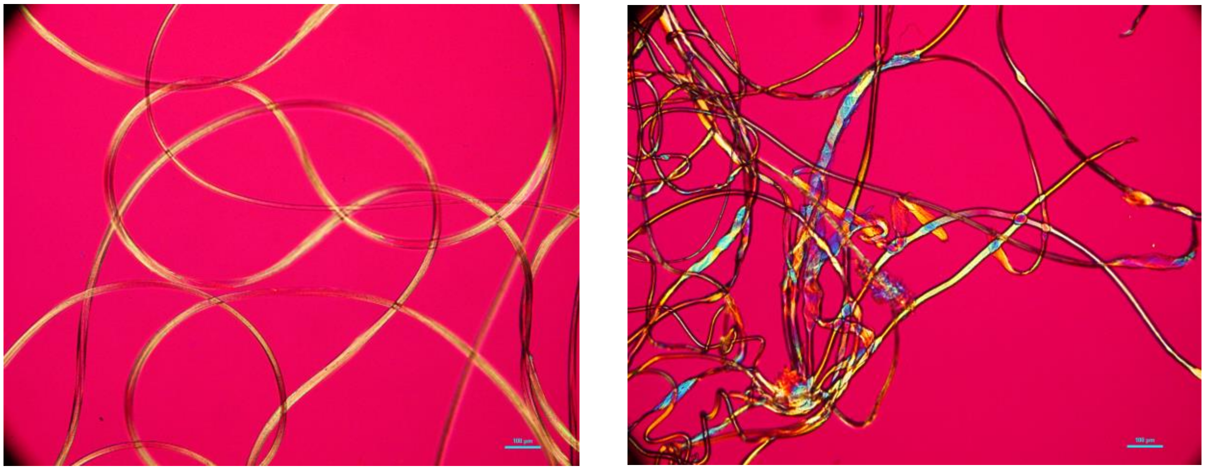

Figure S4. 100x PLM images of FG-3. a) outer cover material from top side of mattress showing homogeneous, birefringent synthetic fibers. b) outer cover material from bottom side of mattress showing a heterogeneous mixture of birefringent synthetic fibers. The scale bars are 100 μm long.

(a)

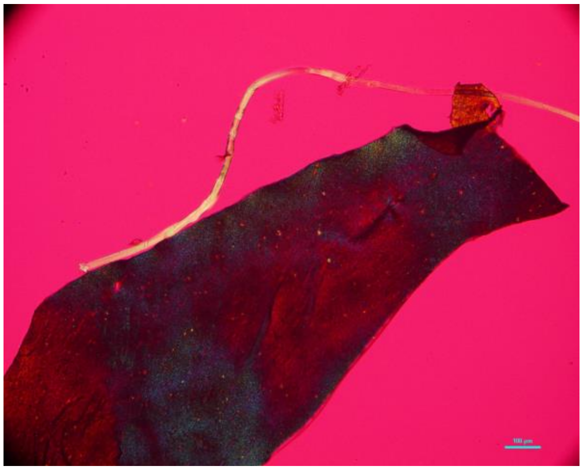

(b)

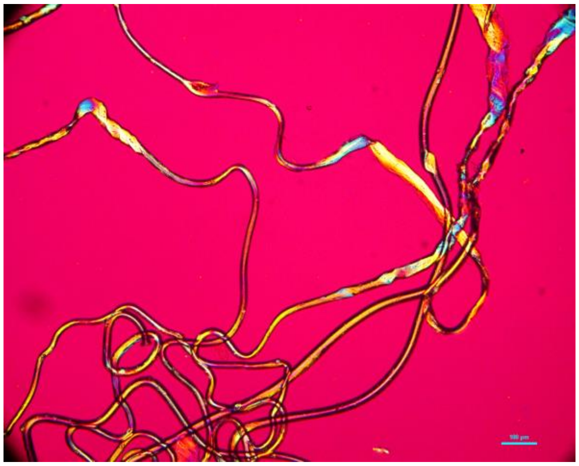

(c)

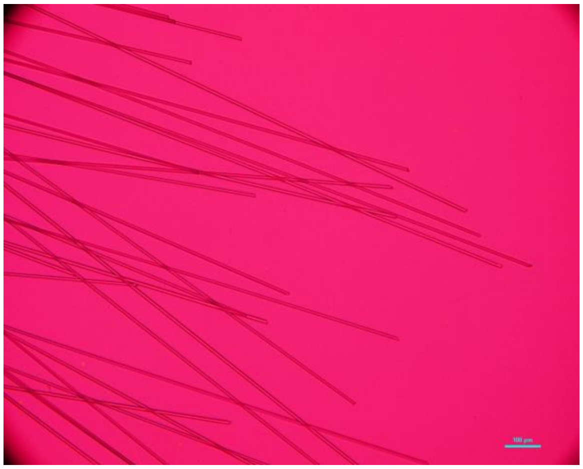

Figure S5. 100x PLM images of FG-4. a) outer cover material showing homogeneous, birefringent synthetic fibers and semi-opaque plastic film. b) inner cover material showing a heterogeneous mixture of birefringent synthetic fibers. c) inner sock material, showing straight, isotropic fiberglass fibers with blunt terminations. The scale bars are 100 μm long.

(a) (b)

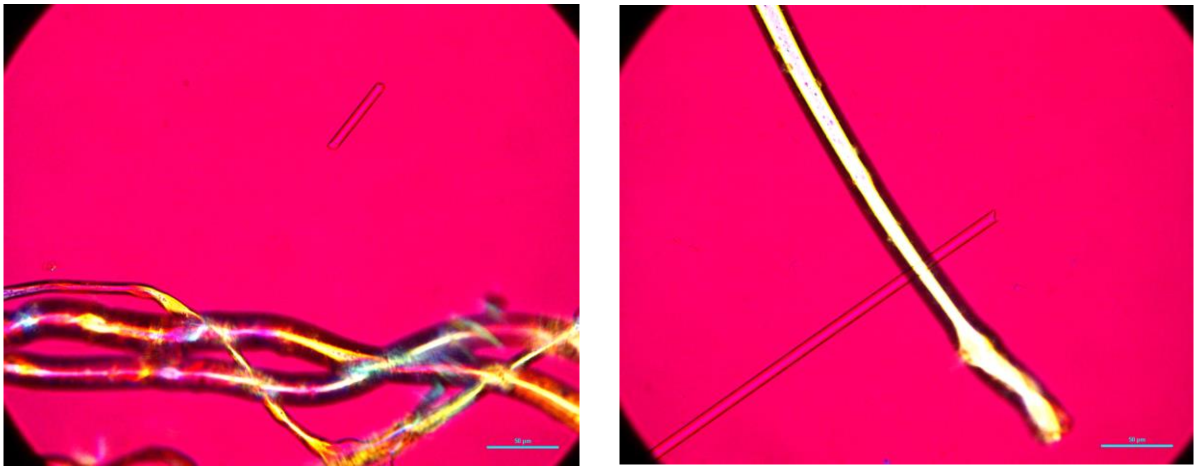

(c)

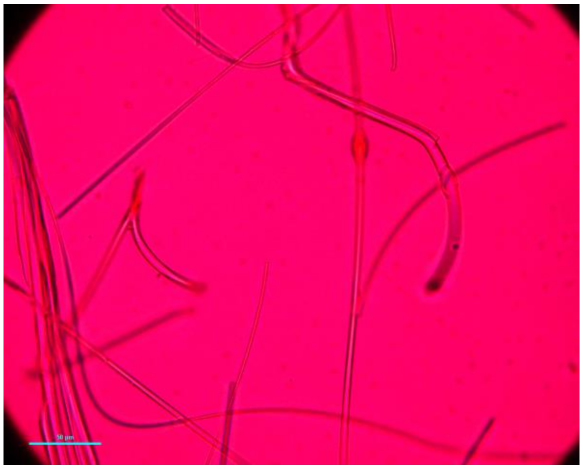

Figure S6. 400x PLM images of fiberglass morphology from a) FG-3 outer cover from bottom side b) FG-4 inner cover c) NIST fiberglass SRM. The scale bars are 50 μm long.

(a)

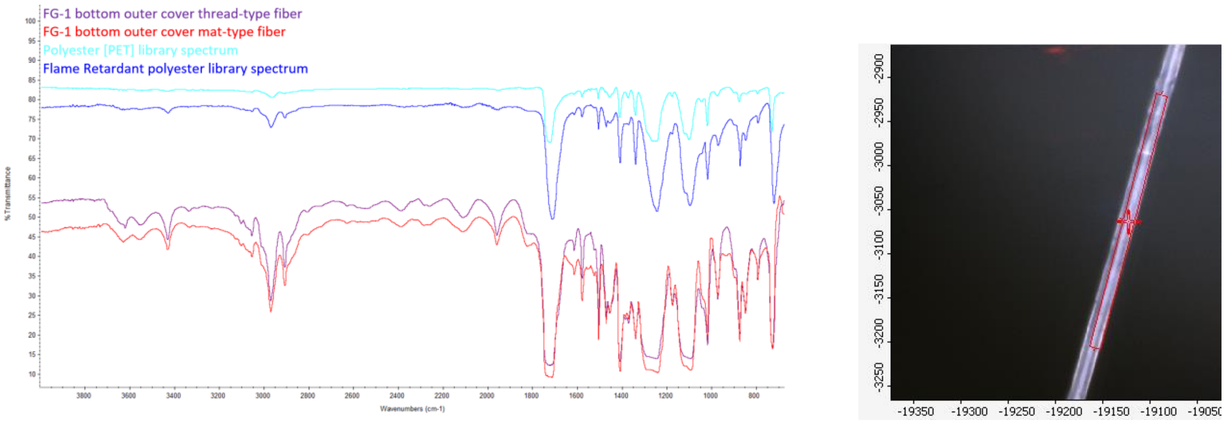

(b)

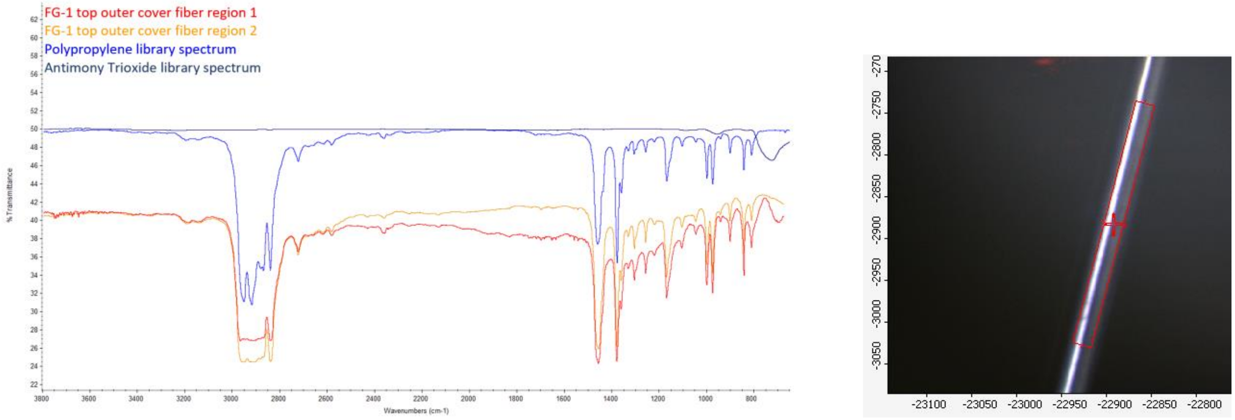

(c)

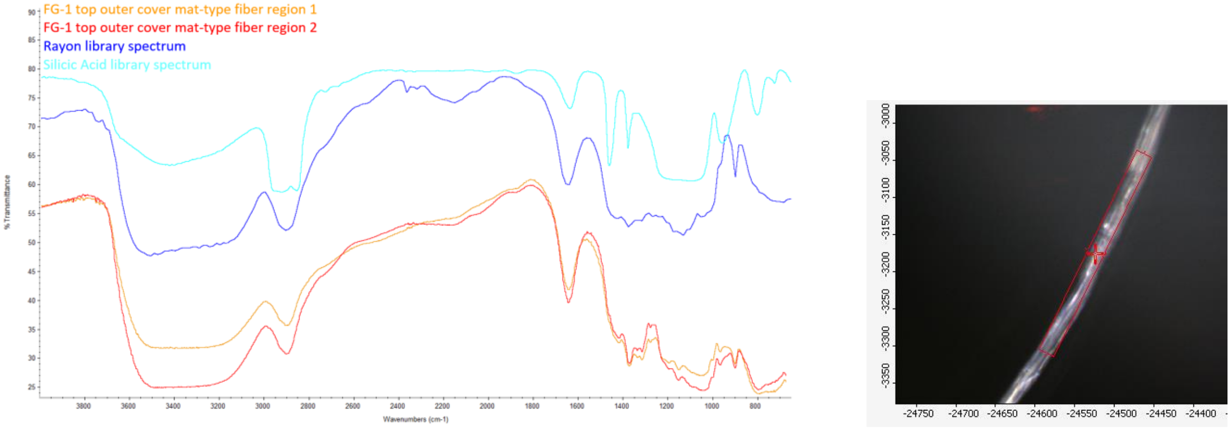

Figure S7. FTIR from three types of FG-1 fibers (red, orange, and purple spectra) and spectral library matches (blue and light blue): a) Flame retardant polyester and unmodified polyester. b) Polypropylene and antimony trioxide. c) Viscose (Rayon) and silicic acid.

Table S1. Mattress labels, certifications, and cover fibers identified.

ID	Mattress	Components listed on flammability label	Chemical certifications and labels	Other labeled components	Cover fibers identified in this work
FG1	Sealy Essentials Joyfulness 8.5" Firm Mattress - Twin	polyurethane foam, Rayon, polyester	CertiPur-US*		Polyester, polypropylene, and Rayon (possible antimony trioxide and silicic acid)
FG2	Modway Aveline 6" Twin	polyurethane foam, viscoelastic polyurethane foam	CertiPur-US	Outer cover top panel: polyester<br>Outer cover bottom: polyester, cotton<br>Inner cover: cotton	Synthetic and cellulosic (PLM only)
FG3	Zinus Green Tea 6" memory foam	polyurethane foam, viscoelastic polyurethane foam	CertiPur-US*	Outer cover top/border: polyester<br>Outer cover bottom: polypropylene<br>Inner cover: fiberglass, acrylic	Fiberglass, flame retardant modacrylic/antimony trioxide, and polyester
FG4	Graco crib & toddler deluxe mattress	polyurethane foam	CertiPur-US*, SB 1019 tag**	Fiber content: polyester	Fiberglass and flame retardant modacrylic/antimony trioxide

* advertised online but not on product tag

** label specified by California flame retardant laws (BHGS, 2019) with box checked next to the line, "THE UPHOLSTERY MATERIALS IN THIS PRODUCT CONTAIN NO ADDED FLAME RETARDANT CHEMICALS."

Table S2. Summary of compositions determined from mattress cover analyses.

Mattress	Cover component	Composition (% by mass)				
		Fiberglass	Cellulosic fibers	Synthetic fibers	Adhesives/ Binders	Other non-fibrous
FG1 Sealy	Top outer cover + mat			80	20	
	Side outer cover			85	15	
	Bottom outer cover			80	20	
FG2 Modway	Top outer cover			100		
	Side outer cover			100		
	Inner cover		100			
	Bottom outer cover		5	95		
FG3 Zinus	Top/side outer cover			99		1
	Inner sock	50		49		1
	Bottom outer cover	<1		75	25	
FG4 Graco	Outer cover			85	15	
	Inner cover	1		69	30	
	Inner sock	75		24		1
Blank	EHLB Ref. #281			5		95
Ref. sample	NIST 1866a	100				
